# Engineering anti-cancer nanovaccine based on antigen cross-presentation

**DOI:** 10.1042/BSR20193220

**Published:** 2019-10-18

**Authors:** Vaishnavi U. Warrier, Amina I. Makandar, Manoj Garg, Gautam Sethi, Ravi Kant, Jayanta K. Pal, Eiji Yuba, Rajesh Kumar Gupta

**Affiliations:** 1 Protein Biochemistry Research Laboratory, Dr. D.Y. Patil Biotechnology and Bioinformatics Institute, Dr. D.Y. Patil Vidyapeeth (Deemed to be University), Tathawade, Pune 411033, Maharashtra, India; 2 Amity Institute of Molecular Medicine and Stem Cell Research (AIMMSCR), Amity University, Noida 201313, Uttar Pradesh, India; 3 Department of Pharmacology, Yong Loo Lin School of Medicine, National University of Singapore, Singapore 117600, Singapore; 4 Experimental Neuroimmunology, Klinikum rechts der Isar, Technical University of Munich, Munich, Germany; 5 Department of Applied Chemistry, Graduate School of Engineering, Osaka Prefecture University, 1-1 Gakuen-cho, Naka-ku, Sakai 599-8531, Osaka, Japan

**Keywords:** Antigen Cross-presentation, Cancer immunotherapy, DC-SIGN, Dendritic cell (DC), Glycan, Nanocarriers

## Abstract

Dendritic cells (DCs) present exogenous antigens on major histocompatibility complex (MHC) class I molecules, thereby activating CD8^+^ T cells, contributing to tumor elimination through a mechanism known as antigen cross-presentation. A variety of factors such as maturation state of DCs, co-stimulatory signals, T-cell microenvironment, antigen internalization routes and adjuvants regulate the process of DC-mediated antigen cross-presentation. Recently, the development of successful cancer immunotherapies may be attributed to the ability of DCs to cross-present tumor antigens. In this review article, we focus on the underlying mechanism of antigen cross-presentation and ways to improve antigen cross-presentation in different DC subsets. We have critically summarized the recent developments in the generation of novel nanovaccines for robust CD8^+^ T-cell response in cancer. In this context, we have reviewed nanocarriers that have been used for cancer immunotherapeutics based on antigen cross-presentation mechanism. Additionally, we have also expressed our views on the future applications of this mechanism in curing cancer.

## Introduction

Cancer caused by relentlessly dividing normal cells is a leading cause of death of several individuals. Cancer remediation using conventional strategies such as chemotherapy and radiotherapy, applied at metastatic stages of cancer, were often not effective [1–4]. In contrast, cancer immunotherapy developed during the last decade induces an immune response developed endogenously to specifically control tumor growth. A few anti-cancer vaccines could translate from bench to bedside due to the immune suppression wielded by the tumor [5]. These vaccines often fail to induce an immune response in an already damaged and tumor burdened immune system. Thus, a lot still remains to be done based on our recent understanding of the functioning and regulation of our immune system. Antigen cross-presentation accomplishes a fundamental capacity toward tumor and microbial immunity. The dendritic cells (DCs) exhibit a remarkable ability to phagocytose and present microbial or tumor antigen to CD8^+^ T cells or CTLs (cytotoxic T lymphocytes) [6,7]. This ability of DCs to cross-present is limited to particular subsets. Several research groups have been working on strategies to enhance cross-presentation and thereby improve T-cell response to tumors. Some studies suggest that cross-presentation by DCs may be subjective to the type of antigen and the timing of inflammatory signals, implying that this process is subject to several varying conditions [8]. Thus, there are several questions that still remain to be answered before we could fully harness the potential of cross-presentation for therapeutic vaccines. Various mechanisms and factors impacting antigen cross-presentation have been proposed by cancer immunologists for successful cross-presentation of the tumor antigens. In this review, we aim to cover the mechanisms of antigen cross-presentation and potential factors involved in enhancing antigen cross-presentation. In addition to that, we focus on current developments in cross-presentation based nanocarriers loaded with antigenic peptides for cancer immunotherapy.

## Profiling of antigen cross-presentation

### Basics of antigen presentation

The immune system uses alternate pathways to clear both intracellular and extracellular pathogens. Endogenous antigens (which are produced within the cell) like the peptides generated from endogenous proteins are processed in the cytosolic or endogenous pathway, conveyed to the endoplasmic reticulum and loaded on to major histocompatibility complex (MHC) class I molecules which is then shifted to plasma membrane in the form of a stable peptide–MHC I complex [9]. Meanwhile, the exogenous antigens (which are endocytosed from the extracellular space) are processed in the exogenous pathway and presented on the membrane with class II MHC molecules. Thus the mode of antigen entry into the cells and the antigen processing site determines whether antigenic peptides get presented with class I MHC molecules, recognized by CD8^+^ T cells or with class II molecules subsequently recognized by CD4^+^ T cells [10]. Inside ER, MHC II molecules are stabilized by the invariant chain (CD74), which is also responsible for the proper folding and protection of its peptide-binding groove prior to the initiation of MHC II pathway. This property has been specifically evolved to inhibit the MHC II from binding to peptides predestined for MHC class I. Invariant chain associates with αβ domain of MHC II and consists of sorting signals in its cytoplasmic tail directing the transport of MHC II to the endocytic compartments [11]. In each succeeding endocytic compartment, proteolytic activity surges and the invariant chain is steadily degraded by late endosomal proteases like Cathepsin S and L [12,13]. However, a short peptide fragment class-II-associated invariant chain peptide (CLIP), still occupies the peptide-binding groove preventing any untimely binding of the antigenic peptide. The CLIP is later swapped for antigenic fragments catalyzed by the HLA-DM, a heterodimer of α and β chains [14]. The strongly bound peptide vehicles the MHC II to the plasma membrane where it adopts a stable form owing to the neutral pH conditions. The pathway involved in MHC I antigen presentation is substantially more intricate. Intracellular proteins to be degraded are targeted for proteolysis with the attachment of a regulatory protein, the ubiquitin [15]. The proteasome complex in charge of the extralysosomal catabolism of cellular proteins then cleaves the peptide bonds within its central hollow in an ATP consuming process. The peptides are translocated to the RER through transporter protein TAP (transporter associated with antigen processing) accompanied by the hydrolysis of an ATP [16]. Simultaneously the assembly of the MHC I complex occurs that involves numerous steps and contributions of molecular chaperones assisting the folding of the polypeptide. Calnexin and ERp57 bind to the free α chain to promote its folding [17]. While β_2_ microglobulin binds to α chain, calnexin is released and chaperone calreticulin and tapasin (TAP-associated protein) binds, which brings the MHC I in close proximity with TAP. Further, endoplasmic reticulum aminopeptidase (ERAP), an exoprotease in ER conducts *N*-terminal processing of peptides for optimum binding to the groove of MHC I [18,19]. This antigen processing and presentation process is known as the classical MHC I antigen presentation pathway.

### Antigen cross-presentation

Exogenous antigens do not have access to the cytosol where MHC I pathway operates. These exogenous antigens in the cell’s external environment are internalized into endocytic compartments and displayed on MHC II molecules thereby stimulating CD4^+^ T-cell response. An interesting exception to this phenomenon was first discovered in 1976 when it was shown that female mice [F_1_ (BALB/c × BALB.B)] injected with ample allogenic cells congenic for minor histocompatibility antigens surprisingly resulted in the generation of CTLs that were specific for minor antigens from the graft, and were restricted to host MHC class I molecules [20]. This substantial stimulation of CTL response *in vivo* was termed as cross-priming. Thus ‘cross-priming’ is defined as the activation of naive CD8^+^ T cells by antigen-presenting cells (APCs) that have acquired antigens from another cell. The mechanism of cross-presentation was dubious such that the exogenous antigens would present on MHC I molecule eliciting an immune response that could lead to the death of otherwise healthy CTLs. Despite this anticipation, the cross-presentation phenomenon was confirmed and indeed some unique cells from lymphoid organs when explanted *ex vivo* generated class I presented peptides. A later report showed the depletion of macrophages using silica hence reduced the ability of mice (C57BL/6) to generate CTL response even to live viruses. This indicates that macrophages, in general, may be key components in the initiation of all CTL responses [21]. Further experiments have also demonstrated *in vitro* cross-presentation of exogenous cellular antigens by DCs and macrophages [22,23].

### Regulation of antigen cross-presentation

#### Intracellular pathways governing cross-presentation

**Figure 1:**
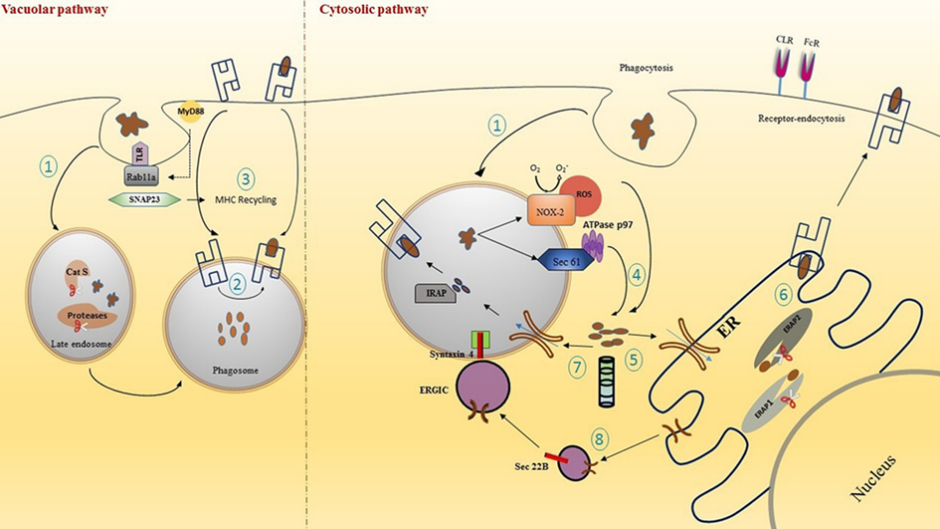
An overview of the mechanisms of antigen cross-presentation Vacuolar and Cytosolic cross-presentation pathways inside a DC: (1) Antigens internalized through phagocytosis or receptor-mediated endocytosis are properly degraded in the endosomes due to varying pH and different proteases. (2) Antigen is loaded on to recycled MHC I in the phagosome. (3) The recycling of MHC I is mediated through Rab11a in Toll-like receptor (TLR) controlled process with the help of SNAP23 dependent on MyD88 signaling. (4) Alternatively, antigens processed under the cytosolic pathway are translocated to the cytosol via phagosomal disruption through NOX-2 complex and through Sec 61 translocon. (5) The antigens are lysed by proteasome complex. (6) Lysed antigens are transported to the ER via TAP transporter and further trimmed by ERAP and loaded on to MHC I. (7) Antigenic peptide in the cytosol after proteasomal degradation may also get retransported into phagosome through TAP which is further trimmed by IRAP and presented on to recycled MHC-I molecule. (8) TAP molecule is transported from ER with the help of Sec22b protein resident of the ERGIC that interacts with Syntaxin 4 on phagosome. Abbreviation: ERGIC, ER-Golgi intermediate compartment.

Extensive studies have shown that peptides from an extracellular antigen are presented on MHC I molecules by a wide variety of mechanisms [24]. In contrast with the classical MHC presentation pathway, the molecular mechanisms that regulate cross-presentation are yet indistinguishable. A number of early studies provide evidence to indicate the occurrence of cross-presentation. An experiment performed by Pfeifer et al. [25], showed that despite inhibiting classical MHC I processing employing Brefeldin A and/or cycloheximide, phagocytosed recombinant *Escherichia coli* antigens were presented on MHC I molecules. Two major mechanisms that have been qualified to further govern the fate of antigen for cross-presentation are as follows (Figure 1): (i) Vacuolar pathway: it is a mechanism wherein lysosomal proteases in the endocytic compartments help to catabolize the proteins which then get loaded on to MHC I molecules. One particular lysosomal protease Cathepsin S is known to play a major role during the degradation of antigen for the vacuolar pathway of antigen cross-presentation. In a study performed by Shen et al. [26], cross-presentation of phagocytosed ovalbumin was found to be TAP independent, and antigenic peptides were generated directly inside the phagosome. This degradation of peptides inside the endocytic compartments was possible owing to Cathepsin S which is preferentially expressed in APCs. It was observed that this TAP-independent presentation was inhibited in cells that are genetically deficient in endosomal protease Cathepsin S [26]. Thus, these experiments validate the critical role of Cathepsin S in the vacuolar pathway of antigen cross-presentation. However, little information is available to substantiate the significance of vacuolar pathway *in vivo* and further investigations are warranted. (ii) Phagosome to cytosol pathway: it is another pathway in which antigens phagocytized into endosomes get transferred to cytosol where proteasome-mediated hydrolysis of antigen occurs. Subsequently, peptides are transported to ER by TAP transporter and get presented on MHC I molecule. Interestingly, it has been noted that some phagosomes also contained TAP molecules [27]. Hence, an alternative mechanism articulates that the antigen-derived peptides after proteasome degradation are transported back into the endosomal compartment where these peptides are trimmed via insulin-regulated aminopeptidase (IRAP) in the endosome instead of ERAP in the ER before loading on to MHC I.

#### Processing and mechanism of antigen transfer from phagosome to the cytosol

The antigenic peptides that get internalized by phagosome are processed before getting loaded on to MHC I. Phagosomes have a relatively hostile acidic environment after engulfment of antigen, which in most cases can completely degrade the antigenic peptides [28]. To avoid such a circumstance, antigens get translocated to the cytosol for their degradation by the proteasome complex. The ultimate question as to how exogenous antigens can cross the membrane barricade between the phagosome to the cytoplasm is yet unsettled. The transport across the endosomal membrane can be aided through an ER-associated degradation (ERAD) pathway which is usually responsible for shifting of unfolded proteins from ER to the cytosol [29–32]. This process is mediated by Sec61 or Derlin-1 and also AAA-ATPase p97/VCP. The presence of ER proteins in the proteome of phagosomes has been proven using mass spectrometry studies performed by Campbell-Valois et al. [33]. A novel function of p97 was established in endocytic trafficking and also regulating the size of early endosomes. Sec61 was shown to express on early endosome and thus it may be playing a role in antigen translocation [34]. The contribution of Sec61 for cross-presentation was determined using its inhibitor Exotoxin A (Exo A). The use of Exo A to inhibit the function of Sec61 had resulted in decreased cross-presentation, emphasizing on an important role of Sec61 for antigen retro-translocation. In succinct, exogenous processed peptide antigens are transported from phagosome through ER-like proteins after penetrating the lipid bilayer in complex with a translocon such as Sec61 or Derlin complex that is involved in the ERAD pathway [35]. Transfer through this protein channel functions with the aid of a cytosolic ATPase p97. A different study has shown the role of another ERAD-related protein p97/VCP for the translocation of a synthetic long peptide to the cytoplasm [36]. An alternative pathway for the transfer of antigen from phagosome to cytosol could be associated with phagosomal disruption. Strong evidence has been given to support this theory that ROS produced by NADPH-oxidase complex (NOX-2) causes a membrane disrupting chain reaction via lipid peroxidation resulting in antigen leakage from phagosomes. This occurs due to the ability of Nox-2 to generate superoxide anions which react with protons resulting in the formation of H_2_O_2_ and other ROS species. These can react with membranes to oxidize the membrane proteins, cholesterol and (poly)unsaturated lipids [37]. Antigen leakage and cross-presentation were visibly suppressed through inhibition of ROS using an inhibitor phenylarsine oxide (PAO); antagonistically these processes were induced when ROS generating photosensitizer was used. However, the direct mechanistic link between NOX-2 originated ROS and cross-presentation is still unknown [38].

#### Transport of MHC I to the phagosome

As mentioned earlier, the processed antigenic peptides may be loaded on to MHC I molecule in the ER or inside the phagosome. To generate peptide–MHC I complex in endocytic compartments, MHC I molecules must be present inside the phagosome. The source of MHC I molecule to be present in the phagosome for antigen loading is broadly dependent on two elementary routes assumed so far: (i) MHC I molecules that have been newly synthesized are transported from ER to endosome and (ii) the functional MHC I on the cell surface are transported to endosomes as part of endocytosis recycling events. A few antigen-loaded and all antigen unloaded MHC class I molecules are conveyed to late endosomes for their degradation. However, some of these could also be re-loaded with antigenic peptides and recycled in the late endosome. The transport of MHC I to endosome for recycling is dependent on Rab11a in a toll-like receptor (TLR)-controlled method and this process is mediated by a SNARE (Soluble NSF Attachment Receptor) protein called SNAP23 (vesicular transport protein) which is dependent on MyD88 signaling [39]. Another mechanism by which MHC I molecules might traffic into endosome is through the invariant chain or CD74. The role of CD74 was confined to the processing of MHC II molecules until a study by Sugita and Brenner [12], which highlighted the association of CD74 with MHC I for trafficking to endosomes. However, the role of CD74 in antigen cross-presentation has not been defined clearly and it is uncertain whether recycling MHC I, like in case of MHC II, requires chaperone proteins such as HLA-DM for peptide exchange. Further, the stability of the unbound MHC I molecule during this mechanism is very low, making it an unfavorable process for MHC I phagosomal trafficking.

#### Conveyance of TAP molecule to phagosome

As discussed earlier, the antigen that has been processed inside the phagosome can be straightaway loaded on to MHC I molecules present in the endocytic compartment. Several molecular types of machinery play a key role in the interaction between ER and phagosome that governs the presence of MHC I in the phagosome [36,40], either due to fusion between ER stacks and phagosomes or with the help of vesicular formations. Sec22b is a SNARE protein which resides inside the ER-Golgi intermediate compartment (ERGIC) and it is essential for the transport of protein cargo from ER to phagosomes. Sec22b has been shown to interact with the plasma membrane SNARE Syntaxin 4 present on phagosome [40]. The above interaction results in a fusion between the two compartments and thus arbitrating the recruitment of TAP along with ER cargo into the phagosome. A study by Cebrian et al. [41] has further validated the above theory by studying the depletion of Sec22b. A knockout of Sec22b was shown to inhibit the recruitment of ER proteins to phagosomes and hence critically compromising on antigen cross-presentation.

### Distinct pathways of antigen cross-presentation

The presence of distinct cross-presentation pathways that are unique from the vacuolar and cytosolic pathways mentioned above have been suggested by a number of reports. In these pathways, the transfer of antigenic peptides are not governed by endocytosis but through direct cell to cell contact. In one of these pathways, a tumor-directed immune response propagated *via* gap junctions between the cells has been detected [42]. The preprocessed tumor antigenic peptides transfer through these channels and enter the normal MHC I pathway. However, this pathway may be restricted in the sense that stability of peptides to be transferred among cells is low. The cells that are presenting the antigenic peptide may not necessarily be APCs but could also be the infected cells themselves. Saccheri et al. [43], in their study have shown that injected bacteria increase the expression of Connexin-43 which is a protein that is usually suppressed in cancer cells. Connexin-43 is responsible for forming gap junctions between the cells and thus efficiently increasing cross-presentation of antigen. Another pathway is the cross-dressing pathway which is similar to the gap-junction pathway where the transfer of closed MHC I molecule is mediated by the cell to cell contact [44]. This implies that the peptide-loaded MHC I can directly move from infected cell to the APC. The exact mechanism for this pathway and its relevance in comparison with other pathways is still not known and further investigations need to be conducted to give us a more detailed understanding of this process.

### DC subsets unveiling antigen cross-presentation

DCs have the most potential among the professional APCs to initiate an adaptive immune response during an infection. Over the last few years, DCs have been nicknamed as ‘natural adjuvants’ owing to their ability to regulate immune tolerance as well as induce immunity. In comparison with macrophages, DC lysosomes have a less hostile environment which may prevent rapid antigen degradation and thus increase the chances of the antigenic peptide to exit to the cytosol for cross-presentation [45]. This phenomenon could be possible owing to active alkalization by NADPH oxidase NOX2 inside the phagosome [46]. The DCs function through three main transport pathways of antigen internalization, which are receptor-mediated endocytosis, phagocytosis, macropinocytosis. The DCs prefer receptor-mediated endocytosis and many receptors are known to govern this preferred mode of antigen internalization such as complement receptors, scavenger receptors, FcRs, C-type lectin receptors (CLRs) and heat shock receptors (HSPs). However, only a defined combination of DC subsets facilitate antigen cross-presentation. Based upon their ontogeny, transcription factors and surface receptors, the DCs have been initially classified into various DC subsets. Majorly in humans and mice, DCs have been subdivided into plasmacytoid DCs (pDCs) and myeloid DCs (mDCs) or conventional DCs (cDCs) [47]. The pDCs are responsible for vascular inflammation and production of type I IFN to combat any microbial infection. Some recent reports have shown that pDCs possess cross-presentation ability towards antigen targeted to specific uptake receptors present on pDCs [48]. However, the antigen cross-presentation role of pDCs has still not been explored widely. In contrast with this, various subsets of mDCs have shown remarkable antigen cross-presentation ability. Although number of mDC subsets are yet to be fully explored, their existence is crucial to decipher our knowledge on antigen cross-presenting cells. In mice, DCs that cross-present express the chemokine XCR1 along with CD8 [49]. The equivalent to this XCR1 in humans was thought to be BDCA3^+^XCR1^+^CD141^+^, which is also very efficient in cross-presentation [8]. CLEC9A receptor on CD8^+^ promotes cross-presentation by recognizing F-actin on necrotic cells and delivers it to appropriate endocytic compartment. In certain conditions such as inflammation, response to cytokines, viral and fungal infections, the XCR1^−^ DCs are the cross-presenting cells and the reason behind this is not fully clear. The ability of DCs to cross-present has been extensively observed in a variety of DC subsets. However, their cross-presenting capability is also simultaneously dependent upon other factors such as antigen routing, its formulation, as well as the signals that play a role in activating DCs to present antigen to CD8^+^ T cells.

### Peripheral strategies applied for enhancing cross-presentation

Harnessing our insights on the mechanism of cross-presentation is fundamental towards strategies to design novel vaccines aimed at creating immunity against cancer and infectious diseases. Refining cross-presentation based vaccination strategy is a promising step towards immune intervention. Two strategies have been implemented to enhance cross-presentation based vaccination. First, many studies have developed drug-based approaches to directly modulate the process *in vivo*. These studies hypothesized that the treatment of chloroquine (medication to treat malaria) could favor cross-presentation through inhibition of endosomal and phagosomal acidification, which protects antigen degradation and their processing through MHC class I antigen presentation pathway. Cross-priming with regard to soluble antigens has been significantly boosted with chloroquine in mice model [50]. In humans, hepatitis B virus (HBV) vaccine responsers boosted with a dose of envelope protein of HBV vaccine in the presence of chloroquine had greatly increased antigen-specific CD8^+^ T-cell response compared with responders who were boosted with choloroquine [51]. A second strategy is to target antigens *in vivo* to DC surface receptors that can mediate endocytosis and show potential CD8^+^ induction for cross-priming. This approach increased CD8^+^ T cell cross-priming to elicit immune response against tumor or infectious diseases but can also cause CD8^+^ T cell cross-tolerance to avoid self-immunity. Antigen targeting to DCs may results in immunity or tolerance based on various parameters such as nature as well as expression of the target, the vector used (antibodies or chaperones or toxin), co–administered adjuvants, formulation of vaccine, as well as targeted region. In mice, a large panel of DC targeting receptors have been used in vaccine-targeting studies but very few among these have been successfully used for human clinical trials as the capacity of human DCs to cross-present is still less explored.

Antigen targeting to DCs is an engaging approach towards future vaccination, but there is still a long way to go before they can be used for clinical applications in human trials. The structural and functional analyses of human DC subtypes, together with the identification of endocytic molecules selectively expressed by these cells, will hopefully allow the development of novel and rational targeted vaccination strategies for induction of immunity in the next few years.

#### Soluble *versus* particulate antigen for enhancement of antigen cross-presentation

Antigens are acknowledged by the DCs either *via* receptor-independent process such as phagocytosis/pinocytosis or *via* receptor-dependent processes. It has been noted that soluble and particulate antigens are processed through dissimilar pathways. The particulate antigens are administered through peripheral ER-like phagosomes that are more prone to exhibit cross-presentation whereas the soluble antigens are headed towards the ER lumen [52]. In comparison with soluble antigen, particulation of antigen could increase immunogenicity by enhancing receptor-mediated antigen uptake [53]. Unlike soluble antigens which are present as synthetic long peptides, the particulate antigens may be presented as necrotic cells or conjugated with antibodies that bind to particular DC receptors. Bacterium-like particles obtained from a Gram-positive bacterium *Lactococcus lactis* was attached to numerous vaccines thus creating a peptidoglycan layer that was shown to increase the induction of CD8^+^ T cells thus showing an increased cross-presentation [54]. Targeting antigens to DC-specific intercellular adhesion molecule-3-grabbing non-integrin (DC-SIGN) using glycan-modified liposomes have shown to prime CD8^+^ T cells in humans thus showing an enhanced cross-presentation rate [55]. Liposome-based carriers have the advantage of being biocompatible, non-toxic and are flexible in terms of size as well as lipid composition [56].

#### Maturation status of DC

Usually DCs present in the peripheral tissues are immature or non-activated. These DCs are responsible for immunotolerance by either T-cell deletion or expansion of suppressor and/or regulatory T cells and they have low cytokine secretion capacity. The DCs get activated or matured when they encounter a microbe leading to differentiation of the antigen-specific T cells into effector T cells. This maturation of DCs is subject to different microbes that express differing PAMP signals to elicit DC sensors or pattern recognition receptor (PRRs). Immature DCs can uptake a broad range of antigens through endocytic mechanism but they are poor at antigen presentation. In contrast, mature DCs have the ability to capture antigens via receptor-mediated endocytosis and get presented on MHC molecules with high efficiency both *in vivo* and *in vitro* [57]. The manipulation of DC maturation using various maturation stimuli has shown high therapeutic potencies. An *in vitro* cross-presentation assay was performed with mature bone marrow DCs in the absence of CD4^+^ T cells silenced by ISS-ODN [immunostimulatory DNA sequences (ISS) structurally defined by CpG motifs found in bacterial DNA and its synthetic oligodeoxynucleotide analogs]. This system revealed the occurrence of cross-presentation induced by TLRs in mature DCs [58]. Topical application of an FDA-approved skin cream Aldara has been shown to enhance migration and maturation of DCs. TLR7 agonist imiquimod was used as an adjuvant in the skin cream for activating DCs in their complex tumor microenvironment. Aldara-stimulated DCs also showed enhanced cross-presentation of a melanoma antigen MART-1, thus increasing specific CD8^+^ T cells [59].

#### Administration of adjuvant along with antigen

APCs have to be activated by several danger signals such as DAMP or PAMP to stimulate necessary co-stimulatory molecules like CD80 or CD86 for T-cell activation. Peptide-based antigens require an adjuvant to generate these danger signals as they are anergic and have poor immunogenicity. Adjuvant serves by inducing DAMP or PAMP that in turn activates various PRRs such as TLRs, CLRs and retinoic acid-inducible gene I (RIG-1) like receptors [60]. These adjuvant components basically trigger cross-presentation by DC maturation [61]. The choice of adjuvant to be used also varies as each DC subset expresses a different set of the above receptors thus influencing cross-presentation. A number of adjuvants have been used to boost tumor immunity. These include attenuated strains called Bacillus Calmette-Guerin from *Mycobacterium bovis* and functions by various means such as activation of macrophages, increased expression of cytokines and B7 co-stimulatory molecules [62]. An endosomal TLR 3 distinguishes a viral infection by sensing dsRNA. Another potent adjuvant called Poly I:C (Polyinosinic-polycytidylic acid) was able to activate antigen-specific antibodies and CTL immune responses [63]. In a particular experiment conducted by Fehres et al. (2014) [59], they isolated human Langerhans cells which showed an increase in cross-presentation with TLR 3 ligand poly I:C. Induction of T-cell response was attained with an antigen embedded nanoscale liposome delivered using small synthetic peptide that represented a TLR-5 binding motif of flagellin [64]. However, thus far adjuvants have shown only a modest range of response for therapeutic treatment of cancer and other infectious diseases [65].

#### Inclusion of cytokines

Large-scale production of cytokines has been possible by isolating and cloning various cytokine genes. Thus recombinant cytokines have also been used as immunostimulators to augment response against cancer. Several groups have constructed recombinant antibody cytokine fusion proteins that show antitumor activity in early phase clinical trials [66,67]. The interferons may act by restoring the expression of MHC molecules on malignant cells lacking these molecules. Similarly, tumor necrosis factor has exhibited direct antitumor property by killing some tumor cells and propagating the inhibition of tumor-induced vascularization [68]. A study by Hoffman et al. [69] indicated that DCs treated with antigen produced more effective antitumor T cells and better cross-presented *in vitro* when they were consequently treated with different cytokines. Another group, Toubaji et al. [70] tested a combination of GM-CSF and IL-2 that when co-administered as an emulsion with a peptide HPV16E7 induced higher CTL and cytokine release responses. However, the difficulty of administering cytokines locally is still an unsettled issue. Another obstacle is the complexity of the cytokine network as the administration of a recombinant cytokine might lead to suppression of immune response [66].

#### Manipulating co-stimulatory signals

Activation of T lymphocyte requires a specific signal in the form of an antigen receptor and also a non-specific or co-stimulatory signal that can attach to ligands expressed on APCs. Absence of a co-stimulatory signal will lead to T-cell clonal anergy or deletion [71]. The lower immunogenicity rates of delivered antigen thus can be attributed to the absence of a strong co-stimulatory signal. Studies have shown that an activating signal such as B7 present on the APCs binds to CD28 on T cells and is essential for their activation [72]. Melanoma cells when transfected with B7 encoding gene, showed differentiation of CTL precursors into effector T cells *in vivo* [72,73]. In recent times, an attempt has been made to treat Glioblastoma multiforme, which is an EGFRvIII-specific third-generation (G3-EGFRvIII) glioma. A chimeric antigen receptor (CAR) was designed that expressed the co-stimulatory factors CD28 and OX40 (MR1-CD8TM-CD28-OX40-CD3ζ) to be targeted on the glioma cells. This demonstrated a prolonged survival of an orthotropic human glioma xenograft model in comparison with the control mice model [74,75].

#### T-cell micro milieu

Several immunological checkpoints are responsible for maintaining tolerance and regulating the expansion of T cells to keep autoimmunity in check. This phenomenon is utilized by the tumor cells in their favor to evade the immune response. Two most studied checkpoint inhibitors, CTL-associated protein 4 (CTLA-4) and PD-1 (programmed cell death 1)/PD-L1 (programmed cell death ligand 1) have been harnessed to aid cancer immunotherapy. In 2018, Nobel Prize was awarded to Dr. James P. Allison and Dr. Tasuku Honjo for their remarkable contribution toward inhibiting the negative immune regulation offered by the checkpoint inhibitors. Initially, FDA approved anti-CTLA-4 monoclonal antibodies (Ipilimumab) were used to block the CTLA-4 pathway to promote antitumor immune response which was the first clinically validated checkpoint blockade strategy [76]. The effect of these antibodies are generated due to blocking the activity of CTLA-4 with its ligand and also F_c_ receptor-mediated depletion of intra-tumoral T_regs_ [77]. Alternatively, when T cells are repetitively stimulated by antigen as in the case of cancer, the level of the inhibitor PD-1 shoots up which leads to a state of exhaustion. Likewise, a range of PD-1 monoclonal antibodies have been constructed such as nivolumab and pembrolizumab for treatments against refractory melanoma and has been approved by the FDA [78]. However, responses to these antibodies have fluctuated on different human trials and efforts had to be made to widen their effects. Thus combinational strategies using checkpoint blockade and other therapeutic approaches have started budding. One such combination therapy involved the usage of an immunosuppressive pathway. Indoleamine-pyrrole 2,3-dioxygenase (IDO) catalyzes the degradation of tryptophan which is essential for optimal T-cell functioning [79]. The tryptophan gets metabolized and is toxic to T cells thus playing a role in immune tolerance. A study performed on this line showed that the combinational usage of drug imatinib decreased IDO expression in myeloid cells along with CTLA-4 antibodies showed an increase in IFNγ production and CD8^+^ T cells [80].

### Therapeutic application of antigen cross-presentation for cancer vaccine development

Fresh insights on immunology have encouraged scientists to develop delivery systems with the purpose of enhancing the induced immune response. Fascinating rationales have been explored for delivering the formulated antigen on to a delivery system. Drug delivery scientists have utilized the underlying mechanisms of cross-presentation to design vaccines of optimum composition. An excellent platform for the timely conveyance of subunit vaccines has been well-thought-out in the form of nanoparticle-coated antigen delivery system. Details of some of these are described below.

#### Vaccines based on targeting of DCs

DCs have been mainly exploited for cancer vaccination through various means. Briefly, three main strategies have been explored: (i) non-targeted peptide/nucleic acid-based vaccines captured *in vivo* by DCs; (ii) vaccine in the form of *ex vivo* generated DCs loaded with antigen (iii) antigen vaccine coupled directly to anti-DC antibodies. As mentioned earlier, the first strategy was based on synthesizing long peptides (25–50 amino acids) potential enough to induce both CD8^+^ and CD4^+^ T-cell responses eliciting a broad range of immunity [81]. Previous vaccines with synthetic peptides were relatively incompetent to induce durable clinical benefit. This leads to the use of long peptides or intact proteins for vaccination and provide strong immune protection because of increased endocytosis, processing and presentation of long versus short peptides [82]. Personalized vaccines can be prepared in lieu of antigen that mimics the tumor peptides from affected individuals owing to novelties in proteomics. However, most tumor-associated antigens of this type are not only less immunogenic but also induce tolerance. Hence these antigens ought to be accompanied by strong adjuvants for generating robust antitumor responses. The second strategy is *ex vivo* generated DC vaccines, where DCs produced *ex vivo* are matured with different antigens and then introduced into affected individuals for treatment. Most immunotherapeutic approaches to date have utilized this strategy of generating *ex vivo* DCs, treating them with tumor-associated antigens combined with adjuvants and then re-injecting for *in vivo* T-cell activation. DCs are produced by *ex vivo* culturing of hematopoietic progenitor cells with different combinations of cytokines. Many procedures have been assessed to make, load and activate DCs *in vitro*. The most common source of DCs for animal studies have been mDCs, derived either from bone marrow precursors or from the blood. These DCs are then activated using cytokines or PRR ligands, loaded with specific tumor antigens. The DCs are administered *in vivo* by tried and tested methods such as intravenous, intradermal or subcutaneous injections [83–85]. Although this was a highly successful technique in terms of patient responses, its major drawbacks were: high costs, laborious and ambiguity in immune response from one person to another. Further, these DCs had reduced migratory capacity due to insufficient maturation. Thus a fairly new technique of developing artificial APCs (aAPCs) has been an attractive and alternative approach [86]. Cellular aAPCs are stable cell lines obtained from genetically modifying xenogeneic or allogeneic cells that can be stored and readily accessed as and when required. Then again, different attempts have been made to synthesize acellular aAPCs which permit a more rigid control of signals for the induction of CD8^+^ T cells through the stimulation of MHC I. Although initial results from these clinical studies have been promising, some aAPCs produced were large, non-deformable and not biodegradable. Therefore, further developments need to be made toward developing aAPCs that can eliminate laborious and costly cell culturing of APCs [87].

#### Antibody-mediated targeting of CLRs

The third approach is targeting DCs *in vivo* making use of the fact that efficient antigen delivery and DC maturation can be propagated by steering the specific receptors on the DCs. Endocytic receptors that naturally detect pathogens have advanced to be promising candidates for targeting of antigen. PRRs present on DCs potentiate them to detect danger signals and triggering these PRRs leads to the uptake of antigen by the DCs for their cross-presentation. Prominent PRR family members include nucleotide-binding oligomerization domain proteins (NOD), retinoic acid-inducible gene 1-like receptors (RIG-I), TLRs and CLRs. The characterization of the biological properties of these PRRs, especially the CLRs can be harnessed to utilize these molecules for targeted antigen delivery. One of the approaches for activation of DC is by the delivery of antigen conjugated antibody against uptake receptor on DC [88]. Specific antibodies are generated and coupled to the antigen of choice. This can be achieved either by chemical coupling between antigen and the specific antibody or by a more expedient method of producing recombinant antibody–antigen constructs. The latter provides an advantage of genetically modifying the targeting antibody for optimal antigen coupling. It also provides the knowledge of the exact number of antigen molecules that can be coupled to the antibody. Another study has shown the use of biotinylated antibodies along with antigen coupled to streptavidin that has been targeted to selected DC subset through β2-integrins or CLRs [89]. The initial study of targeting CLRs was demonstrated by Prof. Ralph Steinman using DEC-205 to elicit CD4^+^ and CD8^+^ antigen-specific immunity *in vivo*. OVA protein-coupled with monoclonal αDEC-205 antibody was presented to CD11c^+^ DCs to elicit this response which was surprisingly 400-times more effective than soluble OVA [90]. This finding paved the way for antigen delivery by the use of anti-CLR monoclonal antibodies with differing potencies toward CLEC9A (DNGR), DCIR-2, DECTIN, ASGPR, mannose receptor (MR) or CLEC12 (DCAL-2). Targeting antigen to the receptor CLEC9A with an OVA-anti-CLEC9A conjugate resulted in cross-presentation and induced CTL responses. In another study by Caminschi et al. [91] targeted delivery of antigen using monoclonal antibodies to DCs against the receptor CLEC9A showed an enhanced CD4^+^ and CD8^+^ T-cell response. These results were also surprising as these responses were seen in the absence of any adjuvants indicating that CLEC9A is a promising target for increasing vaccine effectiveness.

#### Glycan-mediated targeting of DCs through CLRs

Targeting the cell surface receptors on DCs has been a subject of considerable interest. Various receptors that can be targeted on DCs include MRs, DC-SIGN, Scavenger receptor, DEC-205 and TLRs. A type of CLR, such as MR, binds to carbohydrates associated with antigens and then internalize these antigens through phagocytosis. Some of the other CLRs like DEC-205, CLEC9A, Langerin and DC-SIGN direct the phagocytosed antigen toward the cross-presentation pathway. This led to a second strategy which was based on the binding of natural ligands against these receptors. As the CLRs are a large family which consists of specific carbohydrate recognition domains, natural glycans are an excellent source of ligand that can bind to these receptors. Additionally, they also allow easy conjugation to the carrier systems, are less immunogenic and can be produced on a large scale at relatively low costs. They can also be produced synthetically using organic chemicals [92]. Since some CLRs are specific to several glycans, this strategy can be used to target several CLRs simultaneously. The glycan specificity of most CLRs except DEC-205, CLEC9A and DCIR-2 are known and hence this targeting strategy can be prolifically applied. Glycan modification of antigen has been widely studied in the case of DC-SIGN which is a type II transmembrane CLR highly expressed on mDCs. DC-SIGN has high binding capacity toward mannose containing structures, fucose containing structures and Lewis-type blood antigens (Le^x^, Le^y^, Le^a^ and Le^b^) that are present in large amounts in fungal, bacterial, parasitic and viral pathogens as well as host glycoprotein [93]. Targeting of antigen to CLR DC-SIGN with glycan strongly complement the cross-presentation ability of DCs on both *in vitro* as well as animal models [55].

#### Antibody *versus* glycans in CLR targeting

Antibody-mediated targeting has been for a long time the most preferred strategy for the delivery of antigen and *in vivo* targeting of DCs [94]. This strategy has been successful for targeting many CLRs, indicating its efficacy toward CD8^+^ cross-presentation of T cells. However, a major drawback of using anti-CLR antibodies in human trials was the elicitation of immune reactions against these antibodies. Using antibodies for targeting DC receptors could elicit non-specific binding *via* the Fc part and Fc receptor evoking an immune response that may lead to elimination of the therapeutic antibody itself. Alternatively, the emergences of glycan-based approaches for DC targeting have caught the attention of drug delivery scientists worldwide [95]. Glycan-based antigen targeting was more feasible in comparison with antibody-mediated glycan targeting not just in terms of additional specific binding but also due to its flexibility in terms of spatial orientation. With respect to distances between the binding sites of receptor, the spatial orientation of the displayed CLR ligand may be adjusted and carrier systems displaying glycan can be tuned for improved receptor recognition on DCs. Thus in this mode of antigen transport, carriers used for targeting antigen to DC receptors, the spacer that links carrier and carbohydrate ligands may be modified to increase the efficacy of targeting antigen [96] and it is the currently accepted strategy of targeting CLRs for designing vaccines.

## Nanocarrier-based antigen targeting and various approaches used for vaccine delivery

**Figure 2:**
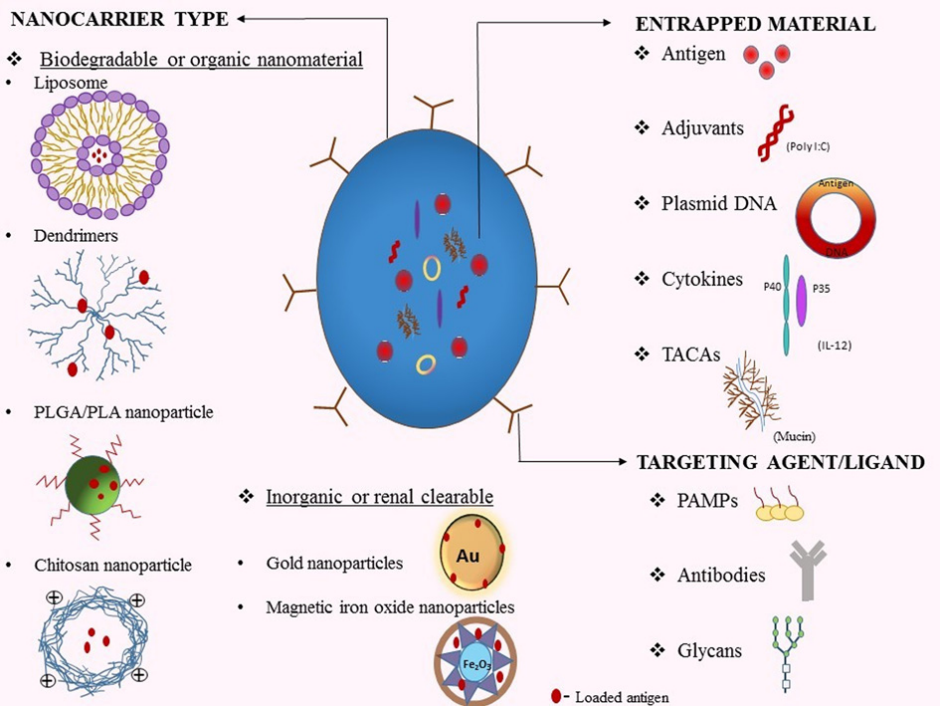
Strategies for designing of nanocarriers employed for the enhancement of antigen cross-presentation: nanocarrier type Different types of nanocarriers with diverse physiognomies have been designed using both organic and inorganic nanomaterials for the delivery of exogenous antigen. Most of the nanoformulations that have been approved for human trials include the organic or biodegradable nanomaterials such as liposomes, dendrimers, polymeric and chitosan particles. Some inorganic nanomaterials that have renal clearable properties such as gold and magnetic iron oxide nanoparticles (IONPs) also have the potential for clinical translation. **Entrapped material**: using nanocarriers for antigen delivery has the advantage that it can club a number of agents like adjuvants, plasmid DNAs, cytokines and tumor-associated carbohydrate antigens (TACAs). These agents need to be directed laterally with the antigen for spiking the response caused by cross-presentation on the immune system. **Targeting agent/ligand**: for the specific targeting of receptors on antigen presenting cells, the antigen loaded nanocarriers can be coated with ligands like antibodies, PAMPs and glycans.

The targeting of antigen toward DCs using a nanoparticle carrier is a broad topic of current interest. Applications of nanotechnology have reformed the field of disease therapeutics. Nanocarrier-based delivery of antigen was developed in order to protect the delivered peptide or nucleic acid from being degraded post-administration. Nanoparticles provide specific antigen targeting platform which can be even more effective and less toxic. The main advantage of using nanoparticles to deliver the antigen lies in its innate ability to protect the antigen. Targeting antigen using this formulation has a higher chance of activating DCs as they more closely resemble pathogens. They can also control the delivery profile of antigen and club multiple numbers of agents presented on their surface that need to be delivered. Widespread research has been devoted toward the co-administration of a number of chemotherapeutics for the fight against cancer. This ability can offer the patient with a multiple co-stimulation using a single nanocarrier. The whole rationale behind designing nanocarrier for antigen delivery is to understand the structural buildup of the nanoparticle and its effect on the immune system. These properties can be defined by the size, charge, shape, surface properties and route of administration of antigen-loaded nanocarriers. A broad range of nanocarrier delivery system has been explored till date including liposomes, dendrimers, polymeric nanoparticles, metallic nanoparticles, virus-like particles (VLPs) [97] (Figure 2). An optimized release profile can be achieved by administering antigen that has been encapsulated, embedded or adsorbed on to nanocarrier. Close to 40 nanoparticulate systems have been approved for theranostic use and still, a huge number of drug–nanoparticulate systems are under clinical and preclinical development phases [98]. However, there is no clear evidence related to the long-term toxicity effects of these conjugates and a lot of further studies need to be performed to assess the same.

### Liposomes

Liposomes as particulate carriers to deliver antigens have unleashed a number of advantages. They have proved to be attractive vaccine delivery candidates as they are derived naturally, are less toxic and fairly accepted by our immune system [99]. Liposomes are stabilized with the help of an inter-bilayer cross-linking and an additional surface charge. They can be targeted through different strategies to enhance the cross-presentation of antigen. Often the cationic liposomes have proven their likely potential for both cellular and humoral immune responses [100]. Antigenic cross-presentation is powerful with liposomes [101]. Similarly, antigens like ovalbumin which are internalized through MR are targeted to early endosomes, which favor cross-presentation. To achieve an increased cross-presentation, liposome-based antigen delivery systems have been rationally designed to retain surface modifications using pH-responsive or fusogenic materials. This strategy leads to cross-presentation of exogenous antigens *via* the cytosolic pathway and alternatively, the targeting of receptors on APCs leading to selective release of exogenous antigen in early endosomes, activates the vacuolar pathway of cross-presentation [102]. Thus, a comprehensive understanding of the mechanisms involved in the induction of immune system can help us in designing targeted liposome-based antigen delivery for cancer immunotherapy. To achieve selective targeting, specific ligands have been implanted at the surface of the liposome. In this respect, their coating with various carbohydrate groups (glycoliposomes) have been in use for several years, exploiting their specificities toward receptors on APCs [103]. A novel strategy has used glycans, the natural ligands of CLRs, that is coated on to liposomes for targeting antigen to DCs. This study by Unger et al. [55], was performed to increase mouse as well as human T-cell responses using Le^b^ or Le^x^ glycan which displayed substantial antigen presentation to CD4^+^ and CD8^+^ T cells. Likewise, when human moDCs were incubated with MART-1 containing liposomes coated with Le^x^ for melanoma treatment, it led to increased antigen presentation to specific CD8^+^ T-cell clones [104]. A pioneering work by Boks et al. [105] have designed an all in one liposome formulation that contained a glycan Le^x^, an adjuvant and a melanoma-associated peptide antigen derived from gp100_280–288_. These glycoliposomes when incorporated with the adjuvant MPLA (monophosphoryl lipid A), a prominent TLR4 ligand, have shown to induce DC maturation as well as produce pro-inflammatory cytokines in a DC-SIGN-specific manner. Thus adjuvant containing glycoliposome vaccine significantly enhanced tumor antigen-specific CD8^+^ T-cell response [105]. Another interesting result was obtained with mannosylated pH-responsive nanoliposomes (MPNLs) which when loaded with OVA showed an increase in interleukins IL-1, IL-10, IFN-γ and serum IgG antibody in BALB/c C57BL/6 mice. It also enhanced cross-presentation to T_h_ CD8^+^ cells, thus affirming the usage of MPNLs as potent antigen targeting vehicle [106]. Yuba et al. [102] have conducted several studies to achieve induced tumor-specific immunity produced using pH-sensitive liposomes. Improved delivery of antigenic peptides were observed by administering these peptides through pH-sensitive fusogenic polymer-modified liposomes. These studies are of important significance due to their ability to deliver the antigen to cytosol through membrane fusion between cytosol and weakly acidic endosome. The liposomes were coated using non-toxic and biodegradable polysaccharides such as 3-methylglutarylated poly(glycidol) (MGluPG) and 3-methylglutarylated dextran, which becomes fusogenic under weakly acidic pH [107]. Alternatively, targeted delivery using pH-responsive dextran derivative, 2-Carboxycyclohexane-1-carboxylated dextran was coated on to liposome. This dextran having hydrophobic spacer groups created strong hydrophobic domains at weak acidic pH of endosomes thus efficiently destabilizing the lipid membrane for delivering antigens that were actively taken up by the DCs and delivered to cytosol resulting at very high levels of IL-12 production [108]. Another fascinating attempt by the same group was performed using hyaluronic acid (HA) based pH-sensitive polymers that target cells expressing CD44, a cancer cell surface marker. These modified liposomes showed release of content into cells expressing CD44 with high efficiency. Similar to this, chondroitin sulfate modified pH-sensitive liposomes were also able to deliver model antigenic proteins into cytosol of DCs which results into cytokine production from these cells which further leads to tumor regression [109,110]. Although liposomes are highly revered nanocarriers, their wide-ranging application for clinical trials with respect to their biodegradability remains to be evaluated and vast scope of extent still remains for the study of completely biodegradable liposomes.

### Dendrimers

Nanocarriers designed for targeting multiple antigen vaccines make it impossible to escape from the immune surveillance and hence increase the efficiency of the targeted antigen. DC-SIGN is a typical transmembrane CLR that is arranged into tetramer groups and a certain flexible neck region which supports its interaction with several glycan configurations. This has led to the design of a multivalent synthetic tool such as the dendrimers for targeting DC-SIGN [111]. Thus dendrimers can be defined as bifurcated molecules consisting of terminal groups to which glycans or antigens can be attached. Moreover, dendrimer approaches displayed that a multivalent display of antigen and particle size enhances cross-presentation by DCs. Glycosylation of dendrimers noticed with increased DC-SIGN-mediated uptake of the molecules which favors CD4^+^ as well as CD8^+^ T-cell responses [112]. DCs express specific set of TLRs which may differently respond to TLR ligand which in turn affect the cross presentation [59]. A novel mannose based antigen delivery system has been designed using polyamidoamine (PAMAM) dendrimer. Mice (C57BL/6) immunized with ovalbumin containing mannosylated dendrimer, with strong DC avidity induced DC maturation and T-cell response *in vitro.* Mannosylated dendrimers were shown to greatly enhance delivery of OVA peptide to MHC I as well as MHC II molecules and show *in vivo* induction of antigen cross-presentation [113].

### Polymeric nanoparticles

Poly (d,l-lactide-co-glycolide) PLGA or Polylactide (PLA) delivery system is fairly well studied and has been used widely for antigen targeting [114]. These particles have been approved by the FDA for parenteral human usage and since have been a favorite for applications in a variety of biomedical fields. PLGA allows for a sustained release of entrapped antigen while it undergoes a gradual degradation through bulk erosion once administered. PLGA/PLA provides large pore size and flexibility in terms of particle size which decides the quality of immune response (cellular response favored by nanoparticle and humoral response favored by microparticles) [115]. These particles have shown to provide an enduring response post single point immunization of antigen entrapped polymer particle [116]. However, this response fails to endure for longer times and they need to be administered along with an adjuvant for improved immunogenicity of antigen and enhanced activation of DCs [117]. Thus, there is a need for designing novel PLGA/PLA particles that have good targeting and controlled release property for long-lasting immunity. These particles, when coated with specific ligands on their surface, have the ability to deliver antigen to APCs and result in an enhanced uptake by the DCs [118]. Vaccine delivery system formulated using double emulsion solvent evaporation method was performed. Using this method, soybean agglutinin microparticles were coated on to PLA nanocarrier providing an enhanced interaction of these particles with DCs via CLRs [119]. This type of controlled and targeted antigen delivery system can be used for improved immunogenicity of weak antigens. A study by Kim et al. [120], have introduced PLGA nanoparticles as potential drug carriers of TLR7/8 agonists. PLGA fabricated nanoparticles were found to be an efficient delivery system for TLR7/8 agonists, which can induce DC uptake of antigen and facilitate lymphatic drainage. Another attractive source of polymeric nanoparticles that have been used widely includes the chitosan nanoparticles. They are an attractive source of biopolymer containing multiple reactive groups and thus chitosan nanoparticles can be coupled with targeting antigen using different reactive groups [121]. This property can be utilized to project nanocarriers possessing varied physicochemical properties like particle size, charge and hydrophobicity. A study performed to investigate the response of tumor-specific CTLs to a therapeutic combination of interferon-γ-inducible protein-10 and melanoma TRP2-specific CD8^+^ CD28^+^ T cells embedded inside folate altered chitosan nanoparticles, showed an inhibition of melanoma cells *in vivo*. The therapy showed a significant increased lifetime of mice cell line B-16 and propagated tumor cell apoptosis [122].

### Metallic nanoparticles

The potential of displaying antigen using synthetic nanoparticles have widely been acknowledged for treatment against cancer [123] and infectious diseases [124]. Several characteristics favor the implementation of metallic nanoparticles for delivery of antigen. These particles are chemically inert, have flexible size and shape and can be simply coupled with proteins on their surface which is highly affined to sulfhydryl groups. Different metallic nanoformulations have been designed for elicitation of immune response including gold nanoparticles (AuNPs), iron oxide nanoparticles (IONPs) [125], silica nanoparticles (SNPs) [126], zinc oxide nanoparticles (ZNPs) [127], carbon nanotubes (CNPs) [128] and more. An interesting finding showed that targeting antigen using AuNPs could induce high level of immune response without the use of any adjuvant. This property of intrinsic adjuvant has been demonstrated by many inorganic nanoparticles. A study reported that calcium phosphate nanoparticles could be used as an alternative for alum as an adjuvant in viral protein immunized mice [129]. Although alum-based nanoparticles were a classic source of vaccine adjuvant they fail to elicit the required CTL triggering effects [130,131]. AuNPs have been shown to be readily ingested by phagocytic mononuclear cells and DCs [132]. A study performed by Ahn et al. [133] demonstrated an effective cross-presentation of antigen (EDB-OVA) embedded AuNP targeted to local lymph node resulting in a high CTL response. Magnetic IONPs have been widely used for drug delivering strategies as they have a large surface area and are biocompatible. Magnetic nanoparticles can be efficiently internalized by DCs [134] and can be tracked *in vivo* using magnetic resonance imaging (MRI) post-administration of the vaccine [135]. In a recent study, IONPs were used as a valuable system to deliver tumor-associated carbohydrate antigens (TACAs) that were less immunogenic, for development of anti-cancer vaccines. The association was supported with the help of hydrophobic interactions in which phospholipid functionalized TACA glycopeptides were coated on to magnetic nanoparticles. The results showed a high release of strong antibody responses resulting in eradication of tumor through complement-mediated cytotoxicity [136]. Thus, it paved a way for the utilization of nanoparticles as a novel method for the display of TACA without the need of any carrier protein.

### VLPs

VLPs are homogeneous, virus particle resembling nanoparticles that have been self-assembled and are non-infectious due to lack of natural genome. Their ability to penetrate cells make them an emerging vehicle for targeted delivery of antigen. VLPs have a certain advantage over other nanoparticles due to their large cargo loading capacities and low toxicities [137]. The cross-presentation ability of VLPs containing tumor antigens have been clearly indicated to induce CTL response processed by DCs, either in their own particulate forms or conjoined with cell debris [138]. VLPs, however, have some limitations with regard to their stability and non-uniform surface [97]. VLPs can be surface modified to bind to a variety of ligands to target different receptors on APCs. Additional work needs to be focused on overcoming these limitations for VLPs to be used for antigen delivery.

An emerging strategy involves using immunotherapeutic strategies in combination with conventional therapeutic modalities. Conventional remedies such as chemotherapy, radiotherapy and surgery have been the most preferred mode of cancer treatment [139–141]. An amalgamation between these conventional techniques and modern cancer immunotherapy techniques might prove to be a crucial linkage for extermination of cancer. Nowak et al. (2006) [142] have reported that using chemotherapy to increase the level at which tumor antigens get cross-presented, can transform the tumor into its own vaccine. They have hypothesized that the huge apoptosis induced by chemotherapy could release pro-inflammatory mediators such as HSPs and IL-6, which promote cross-priming [4,143–146]. However, only a few number of DC-based vaccines have been tested on humans as it is difficult to translate the results obtained from mouse models to humans due to different expression levels of DC receptors and other factors. The cross-presenting capacity of DCs in humans are still not deciphered which thus is a major setback toward not only designing vaccines but also applying them on the clinical front.

## Conclusion and future perspective

The conventional treatments for cancer such as surgery, chemotherapy and radiotherapy have although shown significant improvements but they have their shortcomings in terms of cancer relapse and are not efficient at later stages in both solid tumors and leukemias [147–150]. Cancer immunotherapy is an outstanding alternative therapy that tackles this issue. It involves expending the body’s own immune response by exploiting the mechanism of cross-presentation. However, cross-presentation does not essentially lead to cross-priming. There is a need to change the circumstances in which tumor antigens are cross-presented into a more inflammatory condition. Clearly, a selection of the optimal method for antigen delivery to DC, allowing for effective antigen processing and presentation to T cells, will prove critical to the prospective clinical benefit of these approaches. Several factors that determine the cross-presentation of antigen have been exploited for novel immunotherapeutics. These approaches have been tested with some success in animal models and, to some extent, in human clinical trials. Our acquaintance with understanding the mechanism of cross-presentation is vital for designing vaccines against cancer. The use of nanomaterials toward regulating the administration of cancer antigen-based vaccines for targeting specific immune cells is thus a strategy that has encouraged great outcomes. It has become a widely accepted tool for the delivery of antigen and drugs in a controlled routine. An exploration of the diverse nanocarrier systems and their interaction with immune cells will result in the more rational use of these strategies. Findings by several researchers from their *in vitro* and *in vivo* trials on techniques to improve the cross-presentation of antigens using a variety of nanocarriers have redefined our hopes for a more promising strategy towards cancer immunotherapy. In summary, we can fairly conclude that a number of important questions continue to exist and these questions need to be immediately addressed. Irrespectively, cross-presentation using nanocarriers as a vaccine delivery platform is an exciting method to advance DC-based vaccination strategies for anti-tumor therapy.

## Abbreviations

aAPC: artificial antigen-presenting cell
APC: antigen-presenting cell
AuNP: gold nanoparticle
CLIP: class-II-associated invariant chain peptide
CLR: C-type lectin receptor
CTL: cytotoxic T lymphocyte
CTLA-4: CTL-associated protein 4
DC: dendritic cell
DC-SIGN: DC-specific intercellular adhesion molecule-3-grabbing non-integrin
ERAD: ER-associated degradation
ERAP: endoplasmic reticulum aminopeptidase
ERGIC: ER-Golgi intermediate compartment
Exo A: exotoxin A
HBV: Hepatitis B virus
HSP: heat shock receptor
IDO: indoleamine-pyrrole 2,3-dioxygenase
IONP: iron oxide nanoparticle
IRAP: insulin-regulated aminopeptidase
mDC: myeloid DC
MHC: major histocompatibility complex
MPNL: mannosylated pH-responsive nanoliposome
MR: mannose receptor
PD-1: programmed cell death 1
pDC: plasmacytoid DC
PLA: polylactide
poly I:C: polyinosinic-polycytidylic acid
PRR: pattern recognition receptor
SNARE: Soluble NSF Attachment Receptor
TACA: tumor-associated carbohydrate antigen
TAP: transporter associated with antigen processing
TLR: toll-like receptor
VLP: virus-like particle
